# Supplementary material for: Melatonin Modulates the Microenvironment of Glioblastoma Multiforme by Targeting Sirtuin 1
Source: Nutrients. 2019 Jun 14;11(6):1343. doi: 10.3390/nu11061343 (PMC6627125; doi:10.3390/nu11061343)
Supplement: Supplementary file 1 [file nutrients-11-01343-s001.pdf]

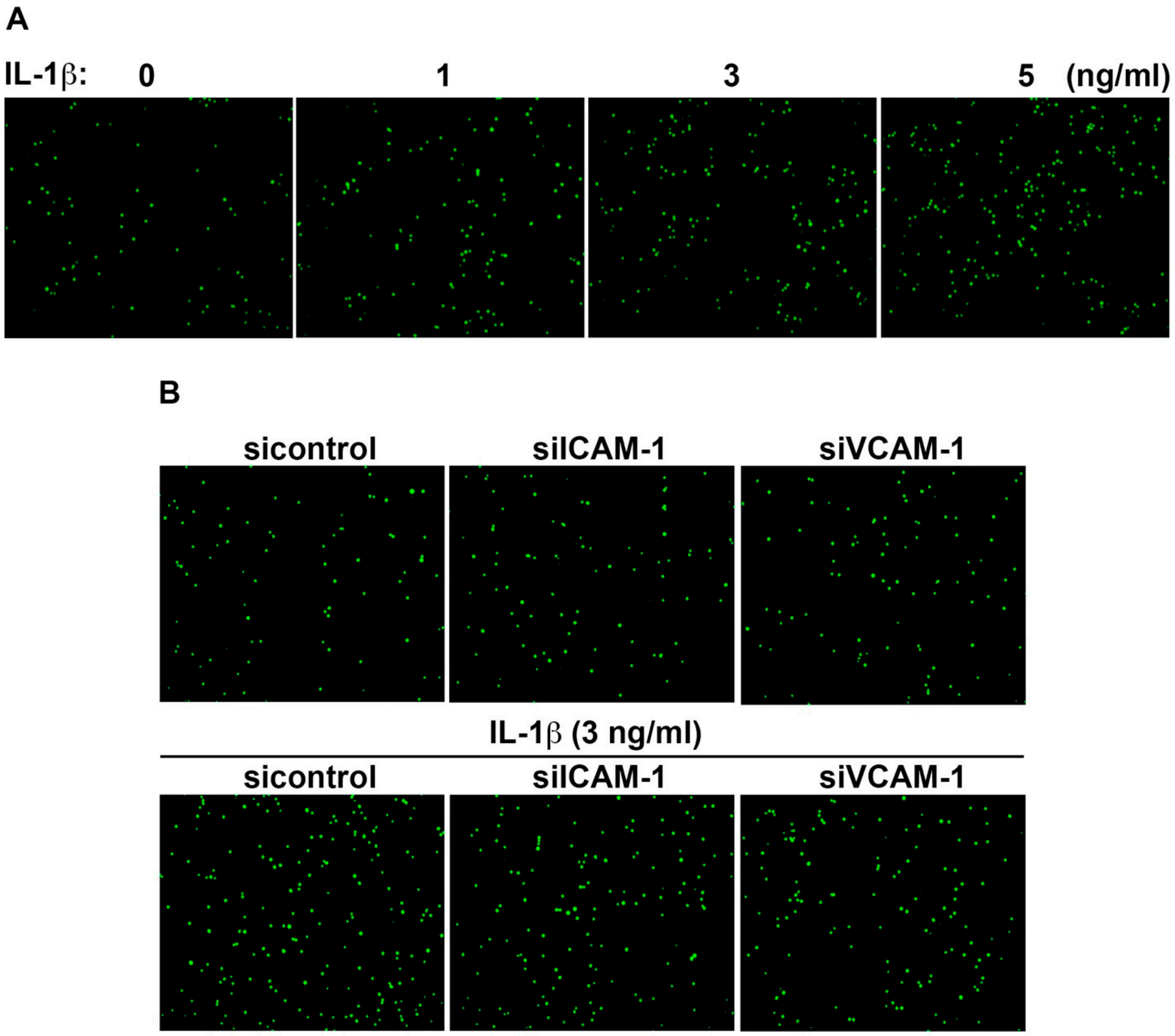

Supplementary Figure 1. VCAM-1 and ICAM-1 regulates IL-1β-enhanced monocyte adhesion to GBM. (A) GBM cells were added with various concentrations of IL-1β (1, 3, or 5 ng/ml) for 24 h. BCECF-AM- labeled-THP-1 were added to GBM cells for 45 min, and then the adherence of THP-1 was analyzed using fluorescence microscopy. (B) GBM cells were transfected with siRNA against control, ICAM-1, or VCAM-1 for 24 h and added with IL-1β (3 ng/ml) for another 24 h. BCECF-AM- labeled-THP-1 were added to GBM cells for 45 min, and then the adherence of THP-1 was analyzed using fluorescence microscopy. The data are presented as mean ± S.E.M (representative of independent experiments = 3).

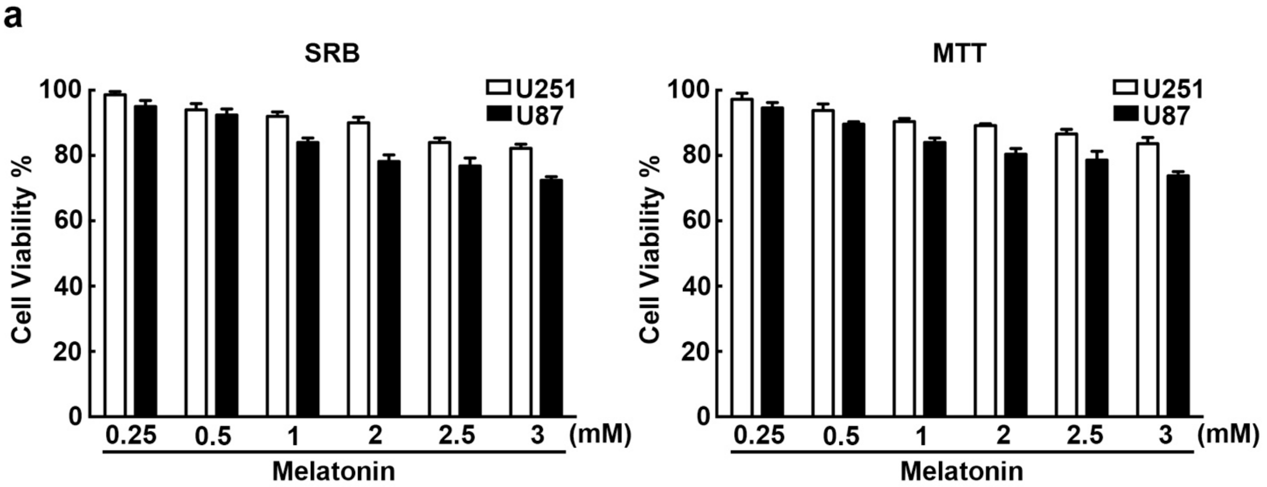

Supplementary Figure 2. Effect of melatonin on GBM viability. GBM cells were treated with various concentration of melatonin (0.25, 0.5, 1, 2, 2.5 and 3 mM) for 24 h. Cell viability was determined using SRB (A) and MTT assays (B) in U251 and U87. Quantitative data are presented as mean ± SEM (representative of $n = 3$).

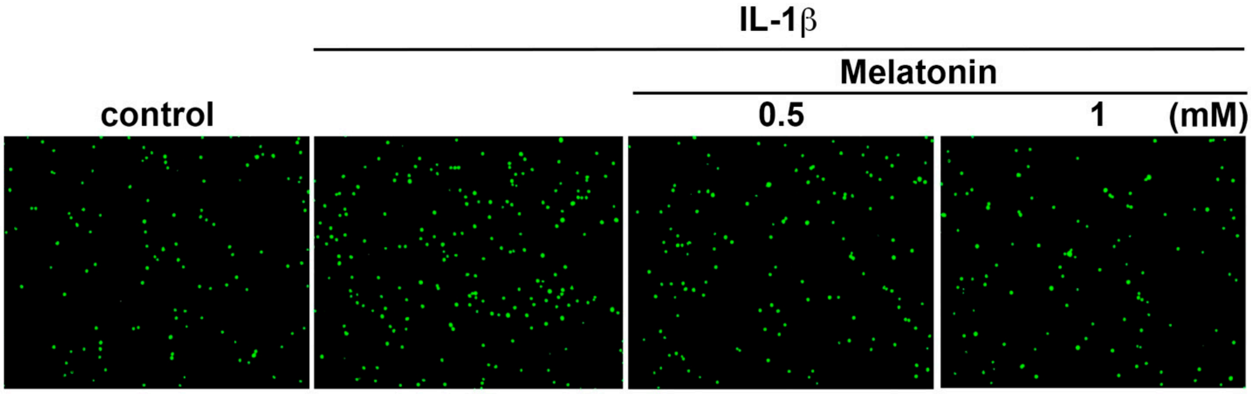

Supplementary Figure 3. Melatonin regulates monocyte adhesion to GBM. GBM cells were pretreated with melatonin (0.5 or 1 mM) for 45 min then added with IL-1β (3 ng/ml) for another 24 h. The BCECF-AM-labeled-THP-1 monocytes by the fluorescence microscopy. The data are presented as mean ± S.E.M (representative of independent experiments =3).
